# High Vaccenic Acid Content in Beef Fat Attenuates High Fat and High Carbohydrate Western Diet Induced Changes in Lipid Metabolism and Gut Microbiota in Pigs

**DOI:** 10.3390/microorganisms9122517

**Published:** 2021-12-06

**Authors:** Vijay P. Singh, Melanie A. Fontaine, Rabban Mangat, Janelle M. Fouhse, Abdoulaye Diane, Benjamin P. Willing, Spencer D. Proctor

**Affiliations:** 1 Metabolic and Cardiovascular Disease Laboratory, Group on Molecular and Cell Biology of Lipids, Alberta Diabetes and Mazankowski Heart Institutes, Edmonton, AB T6G2P5, Canada; vpsingh@ualberta.ca (V.P.S.); rmangat@ualberta.ca (R.M.); abdoulay@ualberta.ca (A.D.); 2 Department of Agricultural Food and Nutritional Science, University of Alberta, Edmonton, AB T6G2P5, Canada; mfontain@ualberta.ca (M.A.F.); fouhse@ualberta.ca (J.M.F.); willing@ualberta.ca (B.P.W.)

**Keywords:** intestinal lipid metabolism, vaccenic acid, insulin resistance, chylomicrons, low birth weight, microbiota

## Abstract

High-fat diets (HFD) have been shown to induce substantial shifts in intestinal microbial community composition and activity which are associated with adverse metabolic outcomes. Furthermore, changes in microbial composition are affected by fatty acid composition; saturated, monounsaturated (MUFA), and industrial trans fats (iTFA) adversely affect microbial diversity while polyunsaturated fats (PUFA) have been shown to have neutral effects. The effects of naturally occurring trans fats on gut microbial composition are unknown. Vaccenic acid (VA) is the most abundant naturally occurring trans fat (abundant in meat and dairy), can be elevated by altering a cow’s diet, and has been shown to have hypolipidemic effects. The aim of this study was to determine how variations of VA content in beef fat affect gut microbial composition, insulin resistance, and lipid metabolism in pigs. Low birth weight (LBW) and control pigs were fed a control or high-fat, high-carbohydrate (HFHC) diet supplemented with beef fat containing either high or low VA levels for 7 weeks. An adapted modified oral glucose tolerance test and fat challenge test were performed at 9 weeks of age following implantation of jugular catheters. Impacts on microbial composition were assessed using 16S rRNA gene amplicon sequencing. The HFHC diet containing beef fat rich in VA had a mild insulin sensitizing effect (*p* < 0.05, slope of curve), increased plasma HDL cholesterol (*p* < 0.05, +28%), reduced postprandial plasma TG (*p* < 0.05), and showed protection from HFHC-induced changes to gut microbial composition in LBW pigs as compared to HFHC diet containing standard beef fat. This is the first study to show effects of natural trans fats on gut dysbiosis; further studies are needed to elucidate mechanisms.

## 1. Introduction

Many studies have demonstrated that a high-fat diet (HFD) can modulate gut microbiota composition and render the gut more permissive to immunogenic bacterial products including lipopolysaccharide (LPS) [1]. In addition to a Western high-fat diet, the composition of fat can also have an impact on gut microbial composition; for example, saturated, monounsaturated (MUFA), and industrial trans fats (iTFA) are characterized by reduced microbial diversity while polyunsaturated fats (PUFA) seem to exhibit a neutral effect on microbial diversity [2]. However, the effects of naturally occurring trans fats on gut microbial composition have not been investigated.

Trans fats are molecules that contain a long hydrocarbon chain with a double bond in the trans configuration. Some trans fats are made synthetically through the partial hydrogenation of vegetable oils and have been widely used in industrial food production, called iTFA [3,4]. Vaccenic acid (VA), the most abundant trans isomer in meat and dairy products, is produced by microorganisms in the rumen through the incomplete biohydrogenation of polyunsaturated fatty acids (PUFAs), α-linoleic acid (ALA), and linolenic acid (LA) [3]. Trans fats have received substantial negative attention over the past decade regarding undesirable health effects [4,5,6]. Currently, trans fat content on a food label does not distinguish iTFA from naturally occurring ruminant trans fats such as vaccenic acid. Industrially produced vegetable fats have been demonstrated to increase risk of coronary heart disease through an increase in circulating total and LDL cholesterol [7]. The structural differences between synthetic and natural trans fats have led to research investigating the specific properties and health effects between the two types of molecules. In particular, vaccenic acid has been shown to have a variety of health benefits including increasing insulin sensitivity as well as anti-inflammatory properties in the intestine [8]. It has also been shown that it is possible to drastically increase the levels of VA in dairy and beef fat by supplementing the diet with flax [9], therefore providing an opportunity to make beef healthier for the consumer.

We have previously shown that low birth weight (LBW) pigs fed a Western diet (high fat, high fructose, and cholesterol; HFHC) develop metabolic complications (upregulate intestinal triglyceride absorption and secretion, develop dyslipidemia and muscular steatosis), display early signs of insulin resistance (IR), and induce changes in gut microbial composition relative to their LBW littermates on control diet [10]. For this study we hypothesized that HFHC diet made with beef fat rich in VA (1.7% *w/w* of diet or 10% of the fat) would protect the host from HFHC diet-induced microbial alterations and improve lipid metabolism relative to standard beef fat.

## 2. Materials and Methods

### 2.1. Animal Housing and Ethics

Piglets were obtained from the Swine Research and Technology Center (SRTC), Department of Agriculture, Food and Nutritional Science, University of Alberta, Canada. All procedures were approved by the University’s Animal Care and Use Committee—Livestock (ACUC) that follow guidelines from the Canadian Council on Animal Care (CCAC) (AUP00001184). Piglets used in the study were Duroc X Large White/Landrace cross. All food, water, temperature, and routine care were provided by trained staff in accordance with animal ethics guidelines. From birth, piglets were weighed weekly, feed intake was measured daily (once housed individually), and each piglet was socialized at minimum of twice weekly to ensure a low stress response during experimental collection.

### 2.2. Study Design

A mean litter weight and standard deviation (SD) was determined to find a 95% confidence interval (CI), categorizing piglets as LBW (less than the 95% CI) or Control (within the 95% CI). Newborn male piglets (*n* = 6 Control and *n* = 16 LBW) (Landrace-Large White × Duroc) were selected and weighed within 24 h of full-term birth from a total of 6 sows. Parity structure was as follows: sow #1: NBW *n* = 1, LBW *n* = 1; sow#2: NBW *n* = 2, LBW *n* = 2; sow#3: NBW *n* = 1, LBW *n* = 2; sow#4: NBW *n* = 2, LBW *n* = 4; sow#5: NBW *n* = 2, LBW *n* = 5; sow#6: NBW *n* = 2, LBW *n* = 2.

**Figure 1:**
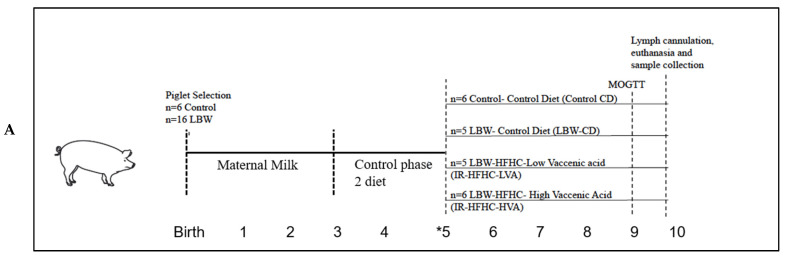

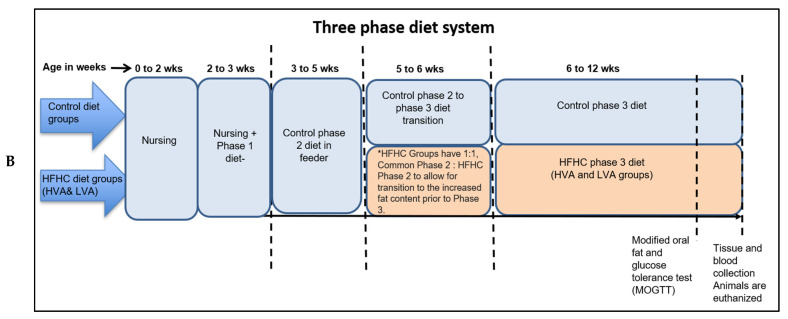
(**A**) Study design schematic: Normal and LBW pigs were fed control, HFHC diet, or HFHC diet supplemented with VA for 5 weeks post-weaning. IR Insulin resistant; MOGTT, modified oral glucose tolerance test; HFHC, high-fat, high-carbohydrate; VA, vaccenic acid. * For HFHC groups, HFHC phase 2 diet (HVA and LVA) partially started at 5 weeks (mixed 1:1 with control Phase 2 diet), completely switched to HFHC phase 3 diets at 6 weeks. (**B**) Three phase diet system: At 2 to 3 weeks of age, pigs are given crumbled solid feed, like giving baby pablum introduced by creep feeder. At 3 to 5 weeks of age all pigs are fed a control phase 2 diet. From 5 to 6 weeks denotes the transition period as described earlier. At week 6 all groups are on respective phase 3 diets. All diets were appropriate for the respective growth stage of the pigs.

Pigs, balanced for litter, were gradually switched onto their treatment group diets (see Table 1 and Table 2 for diets) at week 5 with experimental groups as follows (metabolic type/diet): Control-Control Diet (Control-CD, *n* = 6), LBW-Control Diet (LBW-CD, *n* = 5), LBW fed high-fat, high-carbohydrate, low vaccenic acid diet denoted as Insulin Resistant-HFHC-LVA (IR-HFHC-LVA, *n* = 5) and LBW fed high-fat, high-carbohydrate diet with high VA denoted as Insulin Resistant-HFHC-HVA (IR-HFHC-HVA, *n* = 6). At 8 weeks of age the pigs were fasted overnight and jugular catheters were implanted as previously described [11]. An adapted 2-step modified oral glucose tolerance and fat challenge test (MOGTT) was conducted at 9 weeks of age. Pigs were anesthetized at 10 weeks of age, lymph was sampled, and tissues were collected after euthanasia (Figure 1).

### 2.3. Diet

Pigs were fed diets in a three-phase system as shown in Table 1 and Figure 1. From 2–3 weeks of age pigs were fed a phase 1 diet, which is crumbled solid feed and is introduced by creep feeder. From 3 to 5 weeks of age all pigs were on a control phase 2 diet, fed from feeder. At 5 weeks of age, control diet groups were switched to phase 3 control diet. However, for the HFHC diet groups, the diets were gradually transitioned, to allow the pigs to accommodate to the changing diet. For HFHC groups, HFHC phase 2 diet (respective HVA and LVA diets) partially started at 5 weeks (mixed 1:1 with control Phase 2 diet), and was completely switched to HFHC phase 3 diet at 6 weeks Phase 2 and 3 of the HFHC-HVA diets were made using fat obtained from the Lacombe Research and Development Center, Lacombe, Alberta, Canada. Peri-renal fat was collected from steers fed extruded flaxseed (25%) and hay (75%) sequentially, as previously described [12]. Phase 2 and 3 of the control and HFHC-LVA diets were made with peri-renal fat without VA, which was obtained from a commercial packing plant from steers fed a high barley-grain diet. Methods for fat collection and rendering are as previously described [13]. Fatty acid composition of the diet (Table 2) was determined as previously described using an adapted method [13,14]. The diets were formulated to meet or exceed nutrient requirements of starter pigs [15] (Table 1). Pigs were given ad libitum access to feed and water.

### 2.4. Modified Oral Glucose Tolerance Test

At 10 weeks of age the pigs were fasted overnight and general anesthetic was induced with isoflurane. Under sterile conditions, a catheter was implanted into the left jugular vein allowing for blood collection through a pouch on the back of each swine containing the catheter tubing. Post operatively, each pig was given buprenorphine (0.1 mg/kg every 4–8 h intramuscular for the first 12 h) to decrease pain while trimethoprim-sulpha (50 mg/kg intramuscular) was only given if there were any signs of post-surgical complications as per standard farm protocol [16]. Each catheter was flushed once daily with 1.5 mL to 3 mL (100 IU/mL) heparin in saline solution depending on catheter length [16].

Piglets were fasted overnight at 11 weeks of age and subject to an adapted 2-step modified oral glucose tolerance and fat challenge test (MOGTT). Pigs were weighed and a fasted (time 0) sample of blood was collected via the jugular catheter. Pigs then consumed a 25 g control diet mixed with 1 g/kg body weight Devonshire cream (40% milk fat *w/w*) and 2 g/kg body weight 50% glucose solution. A second meal was given at the 120 min time point containing only 25 g control diet supplemented with Devonshire cream (1 g/kg body weight). Blood was collected at timed intervals (15, 30, 60, 120, 180, 240, 300 min) into EDTA-coated tubes and immediately tested for glucose. Plasma was isolated by centrifugation at 3500× *g* for 10 min at 4 °C and aliquots stored at −80 °C.

### 2.5. Mesenteric Lymph Duct Cannulation and Nascent Lymph Collection

At 13 weeks of age (7 weeks of diet), swine were fasted overnight, weighed and general anesthesia was induced. Anthropometric measurements were again taken as followed at birth. A cannula was implanted surgically into the superior mesenteric lymph duct and lymph was collected for 1 h into an EDTA-coated Vacutainer ^TM^ as previously described [17]. Total lymph volume as well as lymph flow rate (lymph volume/h) was recorded and the animal was terminated via exsanguination under anesthetic immediately upon completion of collection.

### 2.6. Sample Processing

Fasted blood was collected along with tissue samples, including heart, liver, kidney, adipose, muscle, intestine (jejunum and ileum) as well as intestinal scrapings (jejunum and ileum) and snap frozen in liquid nitrogen prior to storage at −80 °C. Prior to freezing, all tissue samples were flushed with ice-cold sterile saline. Blood and lymph were placed on ice until centrifuged at 3500× *g* for 10 min at 4 °C, aliquoted and stored at −80 °C.

### 2.7. Plasma and Lymph Biochemical Analysis

Plasma and lymph were assessed for lipid profiles using commercially available enzymatic colorimetric kits, including triglyceride (TG) (WAKO, Chemicals USA Inc., Richman, VA, USA, Cat#461-08992), glucose (WAKO, Cat#439-90901), LDL-cholesterol (WAKO, Cat#993-00404/999-00504), HDL-cholesterol (WAKO, Cat#993-72593/993-72693), and total cholesterol (TC, WAKO, Cat#439-17501). Glucose was measured at 505/600 nm wavelength while all other parameters were measured at 600/700 nm. Plasma insulin levels were assessed using a commercially available porcine-specific enzyme-linked immunosorbent assay (ELISA) with a detection limit of 0.007 ng/mL and intra-assay coefficient of variation (CV%) of 4.0% at 0.255 ng/mL (ALPCO, Salem, NH, USA) at 450 nm. For lipoprotein cholesterol and TG analysis by fast protein liquid chromatography (FPLC) gel filtration technique, fresh plasma was transferred to Agilent autosample vials and sent for analysis to the University of Alberta Lipidomics core facility with post-column detection of both cholesterol and TG.

### 2.8. Apolipoprotein B48 (apoB48) Quantification

Intestinally derived chylomicron (CM) particle concentration was determined by using an adapted immune Western blotting procedure [18]. Lymph and plasma proteins were separated by SDS-PAGE on a 3–8% Tris-acetate polyacrylamide NuPage gel (Invitrogen, Camarillo, CA, USA). Separated proteins were then transferred to a polyvinylidene fluoride membrane (0.45 μm, ImmobilonTM, Millipore, Billerica, MA, USA). A goat polyclonal antibody specific for apoB (Santa Cruz Biotech, Santa Cruz, CA, USA) was incubated with the membrane overnight at 4 deg, which has specificity for both apoB100 and apoB48 isoforms. An anti-goat secondary antibody tagged with hydrogen peroxidise (Santa Cruz Biotech, Santa Cruz, CA, USA) was used (incubation time 1 h at room temperature) for detection purposes and Enhanced Chemiluminescence (ECL) (Amersham Biosciences Little Chalfont, Bucks, UK) intensity was used to quantify and compare with a known mass of purified rodent apoB48 protein.

### 2.9. Muscle, Liver and Intestinal Lipid Analysis by High Performance Liquid Chromatography (HPLC)

Tissue TG content was assessed by high-pressure liquid chromatography (HPLC). Tissue samples were homogenized using a standard buffer of 250 mM sucrose, 50 mM Tris, 1 mM EDTA with a pH of 7.4 and an added protease inhibitor cocktail tablet (Roche Diagnostics, Germany) using a polytron. Lipids were extracted using the modified Folch method [19] and quantified by HPLC using phosphatidyldimethylethanolamine (PDME) as an internal standard at the Faculty of Medicine and Dentistry Lipid Analysis Core as previously described [20].

### 2.10. Cecal Bacterial Community Characterization

Total DNA was extracted from cecal digesta using the QIAamp DNA Stool Mini Kit (Qiagen, Inc. Germantown, MD, USA) according to the manufacturer’s instructions, with the addition of a bead-beating step to lyse gram positive bacteria (FastPrep instrument, MP Biomedicals, Solon, OH, USA). DNA concentration was measured using a Quant-It™ PicoGreen^®^ dsDNA Assay Kit (Thermo Fischer Scientific, Waltham, MA, USA). Extracted DNA was amplified targeting the V3-V4 regions of the bacterial 16Sr RNA genes using KAPA HiFidelity Hot Start Polymerase (Kapa Biosystems, Inc., Wilmington, MA, USA) with the following conditions: 95 °C for 3 min, followed by 25 cycles of 95 °C for 30 s, 55 °C for 30 s, and 72 °C for 30 s, and 72 °C for 5 min with universal primers (Forward 5′-TCGTCGGCAGCGTCAGATGTGTATAAGAGACAG-3′ and Reverse 5′-GTCTCGTGGGCTCGGAGATGTGTATAAGAGACAG-3′) for amplification [21]. After amplification, AMPure XP beads were used to purify the 16S amplicon to remove primers and primer dimer species. Dual indices and Illumina sequencing adapters were attached using the Nextera XT Index Kit, followed by a second PCR cleanup and quantification. Samples were diluted to 4 nM and 5 μL aliquots of each diluted DNA sample were pooled and size-selected and denatured with NaOH, diluted to 4pM in Illumina HT1 buffer, spiked with 2-PhiX and heat denatured at 96 °C for 2 min. The library was sequenced using a MiSeq 600 cycle v3 kit on an Illumina MiSeq platform (San Diego, IL, USA), according to the manufacturer’s instructions.

### 2.11. Sequence Data Analysis

Sequence data were analyzed using a QIIME2 pipeline (MacQIIME v2019.4) [22]. The Dada2-plugin was used for quality filtration and to generate feature tables; reads were truncated where average quality fell below 20 [23]. Amplicon sequence variants were aligned with MAFFT to construct a phylogenetic tree [24]. Greengenes reference database was used for taxonomic classification of bacterial 16S rRNA gene sequences [25], with amplicons for the domain of interest extracted using primer sequences targeting the V3-V4 regions of the 16S rRNA gene [26]. The R package, phyloseq, was used to visualize the changes in microbial community alpha diversity using Chao1, Shannon, and Simpson indices and the overall microbial community structure using the Bray–Curtis dissimilarly and principal coordinate analysis (PCoA) (R, v3.6.1).

### 2.12. Circulating Endotoxin (LPS) Assay

Serum LPS concentrations were measured using PYROGENT-5000 kit as per manufacturer’s instructions (Lonza, Mississauga, ON, Canada). The absorbance was measured at 340 nm per minute for 1 h at 37 °C using a SpectraMax^®^ M3 Microplate Reader (Molecular Devices, LLC. Sunnyvale, CA, USA). Reaction time was defined as the time required for the absorbance to increase 0.03 absorbance units.

### 2.13. Statistical Analysis

Statistical analysis was performed using the GraphPad Prism 7.02 software. Data were tested for normal distribution using the Shapiro–Wilk test due to its ability to test normality with a lower sample size. One-way ANOVA was used to detect differences between Control-CD and IR-CD, IR-CD and IR-HFHC, as well as IR-HFHC and IR-HFHC + VA treatment groups. Multiple comparisons were tested for using Fisher’s least significant difference (LSD) post-hoc analysis. Pair-matched values of each parameter at each time point for the postprandial curve was also analyzed for significant differences. Results are expressed as means ± SEM. Significance was set using a level of *p* value < 0.05 for all analyses.

Differences in bacterial community structure between treatments was compared using ADONIS. Alpha-diversity measures and relative abundances were compared using nonparametric Wilcoxon rank sum test with npar1way with pairwise multiple comparison analysis achieved using Dwass, Steel, Critchlow-Flingner multiple comparison procedure (SAS Studio University Edition, SAS Institute Inc., Cary, NC, USA).

## 3. Results

### 3.1. Vaccenic Acid Enriched HFHC Diet Increase Plasma HDL-C in Pigs

Low birth weight groups weighed less (*p* < 0.05) and had reduced abdominal circumference than the Control group at birth (*p* < 0.05). Despite weighing less at birth, the low birth weight groups had greater fractional growth rate compared to the control group (*p* < 0.05) (Table 3). At the end of the experiment, there was no difference in the body weight of HVA group compared to the control group (control-control diet), however the LVA group weighed significantly less and had reduced abdominal circumference compared to the control group (control-control diet) (*p* < 0.5). At birth, ‘snout to crown’ ratio was significantly lower in the LVA group than the control group (*p* < 0.05) however at the end of the study, both VA groups had lower ‘snout to crown’ ratio compared to control (*p* < 0.05). There was no difference in the ‘crown to rump’ ratio at birth but at the end of the study, both VA groups had lower ratio than the control group (*p* < 0.05).

IR pigs on a HFHC diet were observed to have significantly greater concentrations of plasma total cholesterol, LDL, TG, and HDL (Table 4). The only fasting parameter that was different (*p* = 0.03) in HFHC-HVA relative to HFHC-LVA was an increase in HDL-C (+28%). However, this result was not significant when Tukey’s multiple comparisons test was used.

### 3.2. Vaccenic Acid Exherts an Insulin Sensitizing Effect

**Figure 2:**
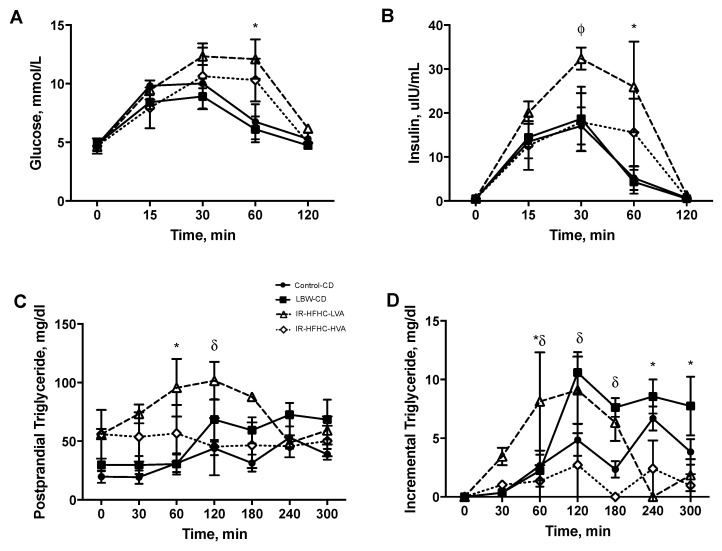
(**A**) Postprandial glucose and (**B**) insulin response following a modified oral glucose challenge. Values are means ± SEM, *n* = 6 Control-Control Diet (CD) (black circles), *n* = 4 LBW-CD (black squares), *n* = 3 IR-HFHC-LVA (open triangles), *n* = 4 IR-HFHC-HVA (open diamonds). (*) denotes statistical difference between LBW-CD and IR-HFHC-LVA (*p* value < 0.05). (ϕ) denotes trend between IR-HFHC-LVA vs. IR-HFHC-HVA (*p* value = 0.09) (**C**) Postprandial triglyceride and (**D**) incremental triglyceride response following a modified oral glucose and fat challenge (MOGTT). Values are means ± SEM, *n* = 6 Control-CD (black circles), *n* = 4 IR-CD (black squares), *n* = 3 IR-HFHC-LVA (open triangles), *n* = 4 IR-HFHC-HVA (open diamonds). AUC is shown (inset). (*) denotes statistical difference between LBW-CD and IR-HFHC-LVA (*p* value < 0.05) and (δ) denotes statistical difference between IR-HFHC and IR-HFHC-HVA (*p* value < 0.05).

HFHC diet, regardless of VA content, significantly increased postprandial glucose concentrations (Figure 2A). Both HFHC diet groups appeared to also have delayed clearance of glucose as both glucose and insulin peak later (30 min) than for control diet (15 min).

Birth weight did not appear to have an impact on insulin response, however, the HFHC-LVA diet significantly increased postprandial insulin concentrations. The HFHC-HVA diet fed to IR swine resulted in a trend towards decreased insulin secretion postprandially without any observed change in glucose AUC (Figure 2B) or HOMA-IR (Supplementary Figure S1).

The slope of the glucose and insulin response was calculated between 0–30 min to represent the initial (appearance) phase and between 30–120 min to represent the second (clearance) phase of the postprandial period (Supplementary Table S1). Based on linear regression, for the initial phase, the slope was significantly increased for both glucose and insulin (*p* < 0.05) in the IR-HFHC group compared to LBW-CD group while the slope was significantly reduced in the IR-HFHC-HVA group (vs. IR-HFHC) but only for the insulin response (*p* < 0.05). For the second (clearance phase), the slope was significantly increased (*p* < 0.05) for insulin response in the IR-HFHC group compared to LBW-CD, however IR-HFHC-HVA diet significantly decreased the slope.

### 3.3. Vaccenic Acid Reduces Postprandial Triglyceride Concentration

When analyzing the incremental curve for postprandial TG response, there are no significant differences in iAUC between control diets or HFHC diet, yet a substantial decrease in TG response was observed when LBW swine were fed a vaccenic acid enriched HFHC diet (Figure 2C,D). There was still significantly reduced incremental TG secretion in the IR-HFHC group compared to IR-HFHC-LVA, when using Tukey’s multi comparisons test (mixed effects analysis), however this was only significantly different at the 60 min time point.

The slope of the TG response was calculated between 0–120 min to represent the initial (secretion) phase of the postprandial period and between 120–300 min to represent the second (clearance) phase of the postprandial period. Based on the linear regression, the slope was significantly decreased in the IR-HFHC-HVA group (*p* < 0.05) compared to IR-HFHC in the initial secretion phase (Supplementary Table S1). In the second clearance phase, the slope was significantly increased in the IR-HFHC group (*p* < 0.05) compared to LBW-control and was decreased in the HFHC-HVA diet.

### 3.4. HFHC Diet in LBW Swine Increases Lymphatic Chylomicron Secretion Rate

On a control diet, LBW pigs had a significant decrease in lymph flow rate (−27%) coinciding with a decrease in lymphatic TG concentration (−55%). No difference between groups was observed with respect to particle size. HFHC diet, irrespective of VA content, significantly increased lymphatic cholesterol levels. Interestingly, no statistical difference was observed in lymph TG content or apoB48 with dietary intervention. However, LBW swine fed a HFHC diet were observed to have a significant increase in apoB48 particle secretion rate (+18%) (apoB48/h) compared to control diet (Table 5).

### 3.5. Tissue Lipid Concentrations

Lipid profiles of three organs, liver, muscle and intestine, were characterized (Table 6). A HFHC diet in LBW pigs decreased liver triglyceride (TG) and free cholesterol (FC), however in the HFHC-HVA group these returned to levels similar to the control diet groups. These results were not observed in the muscle or intestine, with relatively no overall changes between diet and phenotype interventions.

### 3.6. Vaccenic Acid Enriched Diet Attenuates HFHC Diet-Induced Microbial Changes in LBW Pigs

**Figure 3:**
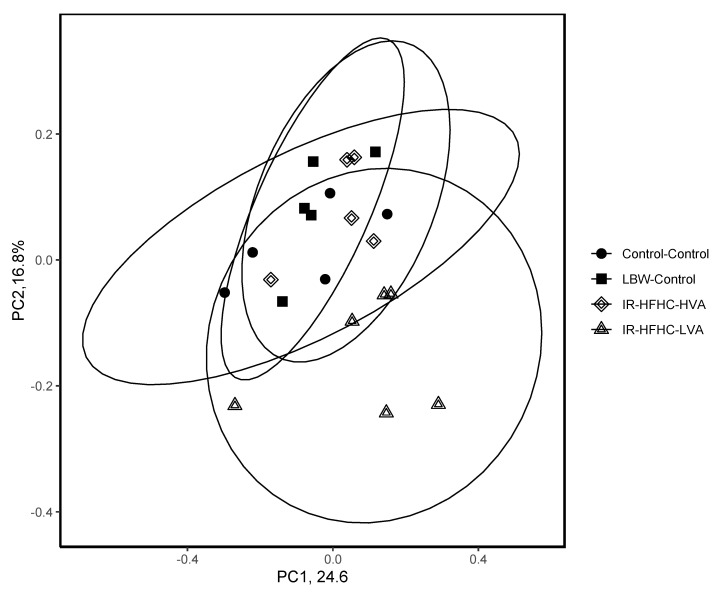
Principle coordinate analysis of cecal bacterial community composition using Bray–Curtis distance metrics. Distinct microbial community structure observed in IR-HFHC-LVA fed pigs was attenuated in pigs fed supplemental vaccenic acid (IR-HFHC-HVA) (ADONIS, *p* = 0.003).

A total of 1,519,492 reads, with an average of 49,685 reads per sample were obtained for a total of 4143 amplicon sequence variants. One sample from the Control-Control group was removed due to low quality sequence data. Principal coordinate analysis (PCoA) of cecal microbiota composition using Bray–Curtis metrics (Figure 3) showed LBW pigs fed IR-HFHC-LVA diet had a significant shift in cecal microbial composition (ADONIS, *R^2^ =* 0.235, *p* = 0.003), while the addition of VA to the IR-HFHC diet normalized the IR-HFHC-LVA diet-induced shift and were not different from pigs fed the control diet.

Cecal microbial composition was impacted by consumption of the IR-HFHC-LVA diet in LBW pigs (Table 7). IR-HFHC-LVA diet intake in LBW pigs resulted in increased relative abundance of Firmicutes (*p* = 0.025) and reduced Bacteroidetes (*p* = 0.022) when compared to control and LBW pigs fed a control diet. The addition of VA to the IR-HFHC diet prevented the decrease in relative abundance of Bacteroidetes in LBW pigs and the increase in relative abundance of Firmicutes, resulting in IR-HFHC-HVA fed pigs having similar microbial composition to control diet fed pigs, regardless of birth weight. At the genera level, addition of VA to the IR-HFHC diet reduced abundance of *Clostridium, Gemminger, Lachnospira*, *Lactobacillus*, and *Roseburia* to similar levels of control diet fed pigs in comparison to those pigs fed the IR-HFHC-LVA diet. The abundance of genera *Parabacteroides* was increased in response to pigs consuming IR-HFHC-HVA relative to IR-HFHC-LVA fed pigs, resulting in similar abundances as control diet fed pigs. The IR-HFHC-HVA fed pigs had the highest documented abundance of *Succinivibrio*, which was significantly greater relative to IR-HFHC-LVA fed pigs. Some taxonomic shifts were unique to the IR-HFHC-HVA fed pigs with *Blautia, RFN20,* and *Turicibacter* abundances being reduced compared to control diet and IR-HFHC-LVA fed pigs.

### 3.7. Species Richness and Evenness of Gut Microbiota

Alpha diversity analysis showed no significant differences in richness or evenness (Chao1, *p* = 0.570 Shannon Index, *p* = 0.240; Simpson Index, *p* = 0.190; Table 8). We also wanted to address if there was an increase in serum LPS in LBW piglets, however we did not detect any evidence for difference between groups. (Supplementary Figure S2).

## 4. Discussion

### 4.1. Effect of Vaccenic Acid on Plasma and Intestinal Lipid Metabolism

There was no consistent catch-up growth in the LBW groups as assessed from zootechnical data. Final body weight measurements indicated catch-up growth except in the LVA group, however this was not recapitulated by the anthropometric measurement data. Another study in miniature LBW piglets demonstrated catch-up growth at 7 months of age, however we used the large swine model and ended the pigs at 10 weeks of age [27].

We observed that LBW pigs fed HFHC diet had higher fasting plasma total cholesterol, LDL, and TG levels compared to pigs fed a control diet. Contrary to previous reports, there were no differences in cholesterol, LDL, or TG concentrations in the plasma in LBW swine fed a HFHC diet enriched in VA compared to those on a HFHC diet with low VA content [7,28]. The most likely cause for inconsistent findings is the use of different models (humans, rodents and pigs) or the form of VA used in these studies (purified VA vs. beef fat). In previous rodent studies [29,30], pure forms of VA supplementation were used in the diet. However, in the current study, LBW swine were fed with beef fat enriched in VA, as opposed to a purified supplement or nutraceutical.

Peroxisome proliferator alpha (PPARa) activation leads to increased plasma HDL [31] and vaccenic acid acts as a Ppara agonist [32]. Consistently, intake of HFHC diet enriched with VA resulted in an increase in fasting plasma HDL. Raising plasma HDL may have a beneficial health effect by reducing cardiovascular event risk and improving glycemic control [33]. The HFHC-HVA diet had a high percentage of cis-9, trans-11 CLA (HFHC = 0.2%, HFHC + VA = 1.28%) which may also be responsible for the increased plasma HDL. Additionally, oleic acid (cis-9 C18:0) has also been known to increase plasma HDL, yet was substantially lower in our HFHC-HVA diet (HFHC = 27.28%, HFHC-HVA = 19.94%) [34]. On the other hand, in a randomized human trial, intake of a VA-enriched diet for 5 weeks resulted in lower total and HDL cholesterol in healthy young men [35], while in another clinical trial, feeding both a CLA- and VA-supplemented diet found no changes in plasma lipids, insulin, or glucose in healthy young men [36]. Since lipoprotein profile in pigs is similar to humans (unlike rodents), our current swine model is a better model to study the mechanisms surrounding these findings for translation to humans. The present findings suggest that feeding IR swine a HFHC diet with beef fat enriched in VA can increase plasma HDL cholesterol, potentially decreasing cardiometabolic risk.

In terms of lymph flow, we found IR swine fed a control diet had lower lymph flow rate and decreased total lymph TG than Control littermates on the same diet. Interestingly, there was no difference in number of CM particles (determined by apoB48 concentration) between metabolic phenotypes (birth weight groups) on a control diet. Our findings suggest that LBW swine fed a control diet secreted less TG in other lipoproteins (such as HDL). There was also no altered lymph flow rate, apoB48, or TG in both HFHC diet intervention (with low or high VA content). Current results are contradictory to previous rodent studies showing decreased TG and CM particle number in lymph (apoB48 concentration) after intake of diet enriched with VA (1% *w/w*) [30]. Current results may be different due to the fact that the diet was previously supplemented with VA in a pure form in the intestine in contrast to this study, which used beef fat enriched in VA. There may be a difference in the bioavailability of each approach.

### 4.2. Insulin Sensitizing Effect of Vaccenic Acid

The VA-enriched HFHC diet resulted in a possible insulin sensitizing effect. VA has been shown to activate peroxisome proliferator-activated receptors (PPARs) and shows insulin sensitizing effects [37]. In this study we found that the VA-supplemented HFHC diet group had no difference in the glucose response (slope for appearance and clearance), however had a less steep slope for insulin response compared to HFHC diet only. In the VA-supplemented diet, we had a 7.87% reduction in palmitic acid (C16:0) content. It has been shown that saturated fatty acid (SFA) (palmitic acid) is associated with LBW [38]. Therefore, lower palmitic acid in the VA-enriched diet may also contribute to the increased insulin sensitivity.

### 4.3. Effect of Vaccenic Acid on Western Type Diet-Induced Gut Microbial Shift

We hypothesized that the LBW-induced increased intestinal lipid absorption may involve a shift in gut microbiota composition [39]. However, the current data did not support this hypothesis, as a phenotype effect (birthweight) was not observed. In agreement with the previous reports on microbiota in pigs, it has been shown that postnatal diet, rather than metabolic phenotype, predominantly shapes gut microbiota composition [40,41,42,43]. Our findings are in accordance with a recent study in low and normal birth weight guinea pigs [44], which showed a significant effect of Western diet on the gut microbiota but no effect of birthweight.

Microbiota composition changes illustrated by PCoA with Bray–Curtis metrics showed IR-HFHC-LVA fed pigs had a significant shift in their cecal microbiota when compared to NBW and LBW control fed pigs. Interestingly, supplementation of IR-HFHC with VA normalized the IR-HFHC-LVA diet-induced shift. Other HFD-induced pig models of metabolic syndrome and obesity have observed similar shifts microbial profiles including enriched Actinobacteria [45], *Lactobacillus* and *Clostridium,* with reduction in Bacteroidetes [43]. While this reduction in Bacteroidetes has previously been noted as a classic alteration in HFD induced obesity, a meta-analysis has revealed there is no association between Bacteroidetes:Firmicutes ratio and obesity risk in people [46]. Inclusion of VA in IR-HFHC pigs abated these characteristic HFD-induced microbial changes back to comparable levels of control pigs. Fat source and composition of high-fat diets play a major role in gut microbial composition and host physiology. A study by Huang et al., 2013, found diets rich in PUFA from safflower oil, versus diets rich in saturated milk fat or lard, significantly influenced microbial structure and increased adipose tissue inflammatory status [47]. Others have shown that fish oil can protect against HFD-induced metabolic disturbances and perturbations in gut barrier function, which is attributed to attenuation of HFD-induced inflammation [48,49]. It is possible that the IR-HFHC-HVA may have reduced the IR-HFHC-LVA induced microbial shift by reducing the expression of inflammatory mediators as well as perhaps other regulatory pathways in LBW pigs. Previous literature demonstrates that blood lipid-lowering and anti-inflammatory effects of VA are partially associated with activation of PPAR-dependent pathway [32]. Our research group demonstrated that VA affects the tissue endocannabinoids (ECs) in JCR:LA-cp rat [8]. Addition of VA increased the concentration of jejunal anandamide and the non-cannabinoid signaling molecules oleoylethanolamide and palmitoylethanolamide. VA treatment also showed decreased expression of two key inflammatory markers, TNFα and IL1β, in the intestine [8], but research is needed to understand the effect of VA on gut microbiota during insulin resistance and related mechanisms.

## 5. Conclusions

In conclusion, using a pig model of metabolic syndrome, we show that a diet supplemented with beef fat enriched with VA has a mild insulin sensitizing effect while raising plasma HDL cholesterol. Supplementation with VA also reduces postprandial incremental plasma TG with unaltered lymphatic TG, CM particle number (apoB48 concentration), and particle size. In terms of gut microbiota, addition of VA to a Western diet showed protection from shifts in composition induced by HFHC diet. More research is needed to confirm the mechanisms of VA on insulin sensitivity and gut microbiota normalization.

## Figures and Tables

**Table 1:** Ingredients of Control and HFHC diets and diet composition of phase 3 Control diet and High Fat, fructose and cholesterol (HFHC) diet.

**Ingredient, g/kg**	**Phase 2**		**Phase 3**	
	**Control**	**HFHC**	**Control**	**HFHC**
Oats	100.00	.	59.00	.
Wheat	463.80	259.40	599.70	229.40
Wheat DDG	30.00	.	50.00	.
High lactose whey	125.00	.	.	.
Soybean meal	168.00	250.00	123.00	230.00
Corn	.	.	94.00	.
Fructose	.	178.00	.	178.00
Fishmeal	37.50	70.00	.	120.00
**Fat (lard) ^a^**	40.00	178.10	25.00	178.10
Flaxseed oil	.	1.90	.	1.90
Cholesterol	.	10.00	.	10.00
Limestone	15.00	8.60	15.00	8.60
Trace mineral swine pre-mix ^b^	.	6.00	.	6.00
Vitamin swine pre-mix ^c^	.	6.00	.	6.00
Salt (NaCl)	0.50	5.00	4.70	5.00
L-Lysine	5.20	9.40	8.30	9.40
Methionine	2.00	5.00	2.70	5.00
L-Threonine	2.20	4.70	3.30	4.70
L-Tryptophan	0.10	0.90	0.40	0.90
Vitamin E—5 KIU/kg premix	1.00	.	0.50	.
Dicalcium phosphate	.	7.00	4.30	7.00
Copper Sulfate	.	.	0.40	.
Others ^d^	9.80	.	9.80	.
**(per 100 g)**	**Control Diet Phase 3**		**HFHC Diet Phase 3**	
Carbohydrate g	55.00		34.00	
Protein g	14.00		21.00	
Fat g	5.00		21.00	
Cholesterol g	0		10.09	
Energy kcal	321.00		409.00	

^a^ Sources of dietary fat: (i) HFHC-HVA diets (phase 2 and 3) were made using fat obtained from the Lacombe Research and Development Center, Lacombe, Alberta, Canada. For this, peri-renal fat was collected from steers fed extruded flaxseed (25%) and hay (75%) sequentially. (ii) Control and HFHC-LVA diets (phase 2 and 3) were made with peri-renal fat without VA, which was obtained from a commercial packing plant from steers fed a high barley-grain diet. ^b^ Provided the following per kg of diet: Zn, 100 mg as ZnSO4; Fe, 80 mg as FeSO4; Cu, 50 mg as CuSO4; Mn, 25 mg as MnSO4; I, 0.5 mg as Ca(IO3)2; and Se, 0.1 mg as Na2SeO. ^c^ Provided the following per kg of diet: vitamin A, 8250 IU; vitamin D3, 825 IU; vitamin 666 E, 40 IU; niacin, 35 mg; D-pantothenic acid, 15 mg; riboflavin, 5 mg; menadione, 4 mg; folic acid, 2 mg; thiamine, 1 mg; d-biotin, 0.2 mg; and vitamin B12, 0.025 mg. ^d^ Provided the following per kg of diet: Vitamin mineral premix, 2.5 g; Bio-mos, 2.0 g; Bioplex zinc 15, 1.70 g; Tetracid 500, 1.0 g; Choline, 0.7 g; Maxi-grow flavor, 0.5 g; Bioplus 2 B, 0.40 g; Superzyme, 0.2 g.

**Table 2:** Fatty acid profiles for major fats in Control and HFHC phase 3 diets using composition of fatty acid methyl esters (FAME).

Abbreviation	Control Diet	HFHC-LVA Diet	HFHC-HVA Diet
**C14:0**	0.09	4.14	3.76
**C16:0**	15.69	30.41	22.54
**C17:0**	0.09	1.30	1.06
**C18:0**	1.67	25.62	21.00
**c9-18:1**	21.34	27.28	19.94
**t11-18:1 (VA)**	n.d.	0.76	10.07
**c9-16:1**	0.21	1.61	1.13
**c11-18:1**	1.77	0.74	0.71
**c9, c12-18:2**	52.87	0.95	1.13
**c9, t11-18:2**	n.d.	0.20	1.28
**t11, c13-18:2**	n.d.	0.00	0.95
**t11, c15-18:2**	n.d.	0.09	2.74
**t13-t14-18:1**	n.d.	0.41	1.58
**C18:3n-3**	4.20	0.20	1.12
Minor Fatty acids	2.07	7.24	10.99
Total	100	100	100

n.d. is used when the percentage of the specific fat was too low to be determined. HFHC, high-fat, high-carbohydrate; LVA, low vaccenic acid; HVA, high vaccenic acid. Minor fatty acids are low abundance or intermediate (odd chain) fatty acids.

**Table 3:** Growth, consumption, and anthropometric measurements of control and LBW pigs fed control, HFHC-LVA, and HFHC-HVA diets until 10 weeks of age.

	**Control-Control Diet**	**LBW-Control Diet**	**IR-HFHC-LVA**	**IR-HFHC-HVA**	***p* Value**
*n*	6	5	5	6	
Birth weight (bw), kg	1.60 ± 0.06 ^a^	1.12 ± 0.17 ^b^	1.14 ± 0.09 ^b^	1.20 ± 0.06 ^b^	0.010
Final weight (fw), kg	39.50 ± 1.32 ^a^	37.80 ± 2.35 ^a,b^	32.90 ± 2.15 ^b^	35.30 ± 0.86 ^a,b^	0.060
ADG, g/d	506.00 ± 3.90 ^a^	495.00 ± 20.90 ^a,b^	443.00 ± 30.00 ^b^	471.00 ± 10.90 ^a,b^	0.128
ADF, g/d	724.00 ± 16.90	718.00 ± 72.00	638.00 ± 46.50	619.00 ± 23.60	0.190
FCR, kg	1.25 ± 0.61	1.28 ± 0.017	1.09 ± 0.02	1.04 ± 0.10	0.080
FGR, (bw − fw)/(d ∗ bw)	324.00 ± 11.50 ^a^	387.00 ± 27.60 ^b^	393.00 ± 21.90 ^b^	397.00 ± 16.50 ^b^	0.040
**Anthropometric Measurements**					
Birth SC, cm	10.60 ± 0.43 ^a^	10.30 ± 0.30 ^a,b^	9.40 ± 0.24 ^b^	9.95 ± 0.20 ^a,b^	0.060
Birth CR, cm	25.50 ± 0.56	24.70 ± 0.64	24.10 ± 0.92	24.30 ± 0.42	0.430
Birth AC, cm	26.80 ± 0.47 ^a^	24.60 ± 0.51 ^b^	24.20 ± 0.64 ^b^	24.20 ± 0.20 ^b^	0.003
End SC, cm	22.80 ± 0.40 ^a^	22.30 ± 0.56 ^a,b^	21.50 ± 0.15 ^b^	21.50 ± 0.22 ^b^	0.050
End CR, cm	83.60 ± 1.11 ^a^	83.00 ± 1.02 ^a^	78.70 ± 1.36 ^b^	78.50 ± 0.74 ^b^	0.003
End AC, cm	72.10 ± 1.15 ^a^	70.80 ± 1.08 ^a,b^	67.90 ± 1.80 ^b^	70.70 ± 0.30 ^a,b^	0.129
Change: SC, cm	13.70 ± 1.44	12.00 ± 0.83	12.10 ± 0.33	11.60 ± 0.24	0.350
Change: CR, cm	58.10 ± 1.23 ^a^	58.30 ± 0.91 ^a^	54.60 ± 1.84 ^b^	54.20 ± 0.77 ^b^	0.040
Change: AC, cm	45.30 ± 1.01	46.20 ± 0.84	43.70 ± 2.09	45.10 ± 1.05	0.630

Values are means ± SEM. LBW, low birth weight; IR, insulin resistant; HFHC, high-fat, high-carbohydrate; LVA, low vaccenic acid; HVA, high vaccenic acid. ADG, average daily weight gain; ADF, average daily feed intake; FCR, feed conversion ratio; FGR, fractional growth rate; SC, snout to crown; CR, crown to rump; AC, abdominal circumference; bw, birth weight; fw, final weight. Change refers to measurement difference from birth to ending. ^a,b^ refer to statistical difference with a *p*-value < 0.05 between different letters.

**Table 4:** Fasting plasma lipid, glucose, and insulin concentrations of control and LBW pigs fed control, HFHC-LVA, and HFHC-HVA diets until 10 weeks of age.

	Control-Control Diet	LBW-Control Diet	IR-HFHC-LVA	IR-HFHC-HVA	*p* Value
***n***	**6**	**5**	**5**	**6**	
**Insulin**, uU/mL	0.32 ± 0.015 ^1^	0.33 ± 0.018 ^1^	0.32 ± 0.010 ^1^	0.31 ± 0.006	0.670
**Glucose**, mmol/L	2.71 ± 0.68	2.70 ± 0.22	2.14 ± 0.20	2.73 ± 0.10	0.720
**Cholesterol**, mg/dL	111 ± 5.82 ^a^	104 ± 8.62 ^a^	239 ± 37.20 ^b^	278 ± 38.20 ^b^	0.003
**LDL**, mg/mL	44.4 ± 1.20 ^a^	43.0 ± 3.80 ^a^	89.5 ± 11.10 ^b^	98.9 ± 10.30 ^b^	<0.001
**TG**, mg/dL	21.2 ± 2.24 ^a,b^	19.0 ± 1.33 ^1,a^	31.2 ± 3.76 ^a,b^	31.4 ± 5.50 ^b^	0.078
**HDL**, mg/mL	26.6 ± 2.67 ^a^	37.2 ± 2.93 ^a,b^	45.3 ± 6.40 ^b^	58.1 ± 3.07 ^c^	<0.001

Values are means ± SEM. LBW, low birth weight; control, control diet; IR, insulin resistant; HFHC, high-fat, high-carbohydrate; LVA, low vaccenic acid; HVA, high vaccenic acid. Sample size is smaller where indicated due to irregularity in analysis and removal using the ROUT method outlier test with a Q value of 10. ^1^ *n* = 4 ^a,b,c^ refer to statistical difference with a *p* value < 0.05 between different letters.

**Table 5:** Fasting lymph lipoprotein and lipid concentrations of control and LBW pigs fed control, HFHC-LVA, and HFHC-HVA diets.

	Control-Control Diet	LBW-Control Diet	IR-HFHC-LVA	IR-HFHC-HVA	*p* Value
***n***	**6**	**5**	**5**	**6**	
**TG**, mg/dL	373.00 ± 50.70 ^a^	168.00 ± 21.70 ^b^	290.00 ± 68.80 ^a,b^	272.00 ± 47.50 ^a,b^	0.071
**Cholesterol**, mg/dL	42.10 ± 3.48 ^a^	40.80 ± 1.40 ^1,a^	68.60 ± 9.52 ^b^	84.70 ± 5.38 ^b^	<0.001
**ApoB48**, μg/mL	273 ± 37.80 ^1,a^	261 ± 50.90 ^1,a^	431 ± 69.90 ^a^	425 ± 73.30 ^2,a^	0.137
**ApoB48/hr**, mg/h	19.79 ± 2.90 ^1,a,b^	11.10 ± 2.60 ^1,a^	29.16 ± 5.70 ^b,c^	36.2 ± 4.90 ^1,c^	0.004
**TG/ApoB48** ratio	1.19 ± 0.29 ^2,a^	0.60 ± 0.04 ^a^	0.95 ± 0.23 ^a^	0.78 ± 0.09 ^a^	0.360
**Particle size**, nm	83.50 ± 1.16 ^1,a^	81.30 ± 6.45 ^a^	93.70 ± 12.0 ^a^	87.90 ± 4.83 ^a^	0.666
**Flow rate**, mL/h	71.70 ± 2.65 ^a^	52.50 ± 4.99 ^1,b^	63.20 ± 6.44 ^1,a,b^	62.40 ± 6.58 ^2,a,b^	0.133

Values are means ± SD. NBW, normal birth weight; LBW, low birth weight; IR, insulin resistant; HFHC, high-fat, high-carbohydrate; LVA, low vaccenic acid; HVA, high vaccenic acid. Sample size is smaller where indicated due to irregularity in analysis and removal using the ROUT method outlier test with a Q value of 10. ^1^ *n* = 4, ^2^ *n* = 5. ^a,b,c^ refer to statistical difference with a *p*-value < 0.05 between different letters.

**Table 6:** Lipid profiles of liver, muscle and intestinal tissue samples of control and LBW pigs fed control, HFHC-LVA, and HFHC-HVA diets.

(μg)	Control-Control Diet	LBW-Control Diet	IR-HFHC-LVA	IR-HFHC-HVA	*p* Value
*Liver* (*n*)	**5**	**5**	**4**	**6**	
**CE 1**	4.93 ± 0.60 ^a,b^	3.03 ± 0.80 ^1,a^	7.69 ± 0.84 ^1,b^	15.43 ± 4.85 ^2,a,b^	* 0.011
**CE 2**	5.58 ± 0.30 ^a,b,c^	4.31 ± 0.53 ^a^	2.42 ± 0.21 ^b^	4.31 ± 0.47 ^a,c^	* 0.020, ** 0.015
**TG**	57.54 ± 18.55 ^a,b^	72.21 ± 8.15 ^a^	17.78 ± 3.83 ^b^	39.40 ± 10.85 ^a,b^	* <0.001
**FC**	5.86 ± 0.48 ^a,b,c^	5.92 ± 0.60 ^a^	3.85 ± 0.30 ^b^	7.49 ± 1.07 ^a,c^	* 0.026, ** 0.028
**FA**	13.29 ± 2.71	23.96 ± 10.67 ^1^	9.91 ± 8.57 ^1^	2.55 ± 0.59 ^3^	
**PE**	30.47 ± 2.12 ^a,b,c^	29.70 ± 3.27 ^a^	19.29 ± 1.74 ^b^	29.61 ± 2.48 ^a,c^	* 0.035, ** 0.016
**PI**	12.32 ± 0.50	13.09 ± 1.53	10.23 ± 1.03	16.54 ± 2.55	
**PS**	8.85 ± 0.59 ^a,b^	8.42 ± 0.72 ^1,a^	5.57 ± 0.47 ^b^	6.19 ± 0.23 ^a,b^	* 0.016
**PC**	47.75 ± 2.86 ^a,b^	49.87 ± 4.64 ^a,b^	37.72 ± 2.65 ^a^	60.11 ± 5.22 ^b^	** 0.011
**SM**	2.55 ± 0.16 ^a^	3.69 ± 0.41 ^b^	4.05 ± 0.88 ^a,b^	4.62 ± 0.34 ^a,b^	# 0.03
*Muscle* (*n*)	**6**	**5**	**5**	**6**	
**CE 1**	-	-	-	-	
**CE 2**	2.02 ± 0.63 ^a,b^	0.49 ± 0.91 ^a^	2.01 ± 0.50 ^b^	1.92 ± 0.42 ^a,b^	* 0.018
**TG**	55.59 ± 19.87	38.52 ± 24.9	55.00 ± 25.24	22.69 ± 7.10	
**FC**	0.62 ± 0.0 ^4^	1.10 ± 0.16	2.07 ± 0.53 ^3^	1.88 ± 0.39 ^3^	
**FA**	8.17 ± 2.16 ^5^	2.80 ± 2.05 ^3^	-	-	
**PE**	10.43 ± 2.46 ^2^	5.80 ± 0.71	9.39 ± 2.47 ^1^	9.61 ± 1.44 ^2^	
**PI**	3.40 ± 0.80 ^1,a^	1.29 ± 0.29 ^1,b^	2.60 ± 1.28 ^1,a,b^	-	# 0.048
**PS**	2.00 ± 0.0 ^4,a,b^	1.76 ± 0.09 ^a^	3.48 ± 0.28 ^1,b^	4.01 ± 0.18 ^a,b^	<0.001
**PC**	17.10 ± 3.5	13.57 ± 1.26	15.02 ± 3.35	16.19 ± 2.61	
**SM**	2.24 ± 0.59	1.16 ± 0.13	1.59 ± 0.19	1.93 ± 0.23	
*Intestine* (*n*)	**6**	**5**	**5**	**6**	
**CE 1**	3.42 ± 1.00 ^1^	3.34 ± 0.265	8.36 ± 2.44	7.07 ± 3.53 ^3^	
**CE 2**	5.09 ± 0.11	5.08 ± 0.16	8.14 ± 2.39	5.59 ± 1.46	
**TG**	44.29 ± 16.88	10.44 ± 1.62	8.47 ± 2.10	37.33 ± 18.71 ^2^	
**FC**	14.68 ± 0.75	13.02 ± 0.7	15.29 ± 4.4	10.58 ± 3.46	
**FA**	21.50 ± 5.03 ^a,b^	17.93 ± 3.49 ^a^	7.39 ± 2.25 ^b^	15.29 ± 7.09 ^a,b^	* 0.035
**PE**	23.80 ± 2.06	21.21 ± 1.18	30.08 ± 7.84	21.81 ± 6.95 ^2^	
**PI**	11.62 ± 0.36	8.65 ± 1.60	12.18 ± 2.95	9.08 ± 2.91 ^2^	
**PS**	11.56 ± 0.69	11.18 ± 0.64	10.99 ± 2.33	7.16 ± 1.61	
**PC**	30.20 ± 3.64	26.39 ± 2.55	44.64 ± 12.00	29.93 ± 10.73	
**SM**	3.24 ± 0.22 ^2^	2.87 ± 0.17	3.90 ± 0.96	3.33 ± 0.80	

Values are means ± SEM. ^a,b,c^ refer to statistical difference with a *p* value < 0.05 between different letters. LBW, low birth weight; control, control diet; HFHC, high-fat, high-carbohydrate; VA, vaccenic acid; CE, cholesteryl ester; TG, triglyceride; FC, free cholesterol; FA, fatty acyls; PE, phosphatidylethanolamine; PI, phosphatidylinositol; PS, phosphatidylserine; PC, phosphatidylcholine; SM, sphingomyelin. Data analyzed using multiple *t* tests. Only significant *p* values are reported: # Control-Control diet vs. LBW-Control diet, * IR-HFHC-LVA vs. LBW-Control diet, ** IR-HFHC-LVA vs. IR-HFHC-HVA Smaller sample size where indicated is due to values lacking during HPLC likely due to small values. ^1^ *n* = 4, ^2^ *n* = 5, ^3^ *n* = 3, ^4^ *n* = 1, ^5^ *n* = 2.

**Table 7:** Relative abundance of differing cecal taxonomy in control and LBW pigs fed control, HFHC-LVA, and HFHC-HVA diets.

Taxonomy	Control-Control Diet ^1^	LBW-Control Diet ^1^	IR-HFHC-LVA ^2^	IR-HFHC-HVA ^1^	*p* Value
**Phyla**					
Actinobacteria	0.07 ± 0.04 ^a,b^	0.04 ± 0.04 ^b^	0.11 ± 0.01 ^a^	0.02 ± 0.01 ^b^	0.022
Bacteroidetes	58.64 ± 6.66 ^a^	55.04 ± 5.72 ^a^	40.73 ± 4.48 ^b^	50.37 ± 2.98 ^a,b^	0.022
Firmicutes	28.13 ± 4.25 ^b^	26.99 ± 4.17 ^b^	46.39 ± 2.24 ^a^	29.69 ± 2.03 ^b^	0.025
**Genera**					
*Blautia*	0.29 ± 0.06 ^a^	0.23 ± 0.05 ^a^	0.17 ± 0.05 ^a,b^	0.04 ± 0.02 ^b^	0.015
*Clostridium*	0.13 ± 0.07 ^a,b^	0.05 ± 0.03 ^b^	2.02 ± 1.00 ^a^	0.27 ± 0.14 ^a,b^	0.035
*Gemmiger*	0.07 ± 0.02 ^b^	0.18 ± 0.04 ^a,b^	0.87 ± 0.24 ^a^	0.42 ± 0.10 ^a^	0.002
*Lachnospira*	0.11 ± 0.05 ^c^	0.13 ± 0.06 ^b,c^	1.24 ± 0.43 ^a^	0.58 ± 0.15 ^a,b^	0.005
*Lactobacillus*	0.84 ± 0.29 ^b^	0.17 ± 0.09 ^b^	4.26 ± 0.57 ^a^	1.74 ± 0.65 ^b^	0.002
*Parabacteroides*	2.01 ± 0.34 ^a^	5.57 ± 1.35 ^a^	0.29 ± 0.13^b^	1.54 ± 0.66 ^a^	0.002
*Peptococcus*	0.01 ± 0.01 ^b^	0.04 ± 0.03 ^a,b^	0.18 ± 0.06 ^a^	0.08 ± 0.01 ^a^	0.006
*RFN20*	0.35 ± 0.10 ^a^	0.50 ± 0.11 ^a^	0.26 ± 0.08 ^a^	0.02 ± 0.01 ^b^	0.008
*Roseburia*	0.69 ± 0.37 ^b^	0.45 ± 0.35 ^b^	3.71 ± 0.77 ^a^	1.35 ± 0.49 ^a,b^	0.008
*Succinivibrio*	3.76 ± 2.21 ^a,b^	3.31 ± 1.75 ^a,b^	1.45 ± 0.65 ^b^	11.07 ± 2.22 ^a^	0.027
*Turicibacter*	0.42 ± 0.22 ^a^	0.17 ± 0.04 ^a^	0.15 ± 0.07 ^a,b^	0.04 ± 0.02 ^b^	0.024

Values are presented as means ± standard error of the mean. ^a,b,c^ refer to statistical difference with a *p* value < 0.05 between different letters. LBW, low birth weight; IR, insulin resistant; HFHC, high-fat, high-carbohydrate; LVA, low vaccenic acid; HVA, high vaccenic acid. ^1^ *n* = 5, ^2^ *n* = 6.

**Table 8:** Alpha diversity of cecal bacteria in LBW and Control pigs fed HFHC and VA-enriched diets.

	Control-Control Diet ^1^	LBW-Control Diet ^1^	IR-HFHC-LVA ^2^	IR-HFHC-HVA ^1^	*p*-Value
**Shannon**	4.87 ± 0.12	4.90 ± 0.22	4.92 ± 0.11	4.67 ± 0.08	0.240
**Chao-1**	399 ± 75	548 ± 184	480 ± 31	356 ± 78	0.570
**Simpson**	0.98 ± 0.001	0.98 ± 0.002	0.98 ± 0.004	0.98 ± 0.003	0.190

Values are presented as means ± standard error of the mean. LBW, low birth weight; IR, insulin resistant; HFHC, high-fat, high-carbohydrate; LVA, low vaccenic acid; HVA, high vaccenic acid. ^1^ *n* = 5, ^2^ *n* = 6.

## Data Availability

The data presented in this study are available on request from the corresponding author. The data are not publicly available due to policies to retain animal ethics protocols.

## Supplementary Materials

The following are available online at https://www.mdpi.com/article/10.3390/microorganisms9122517/s1, Figure S1: Homeostatic Model of Assessment of Insulin Resistance (HOMA IR). Figure S2: Concentration of serum lipopolysaccharide (LPS) in Control and LBW pigs on control diet, HFHC-LVA or HFHC-HVA diet. Table S1: Equations for the (Appearance 0–30 min) phases of the postprandial glucose, insulin and triglyceride curves.

## References

[1] Cani P.D., Amar J., Iglesias M.A., Poggi M., Knauf C., Bastelica D., Neyrinck A.M., Fava F., Tuohy K.M., Chabo C. (2007). Metabolic endotoxemia initiates obesity and insulin resistance. Diabetes.

[2] Wolters M., Ahrens J., Romani-Perez M., Watkins C., Sanz Y., Benitez-Paez A., Stanton C., Gunther K. (2019). Dietary fat, the gut microbiota, and metabolic health—A systematic review conducted within the MyNewGut project. Clin. Nutr..

[3] Wang Y., Proctor S.D. (2013). Current issues surrounding the definition of trans-fatty acids: Implications for health, industry and food labels. Br. J. Nutr..

[4] Stender S., Astrup A., Dyerberg J. (2008). Ruminant and industrially produced trans fatty acids: Health aspects. Food Nutr. Res..

[5] Sarnyai F., Somogyi A., Gor-Nagy Z., Zambo V., Szelenyi P., Matyasi J., Simon-Szabo L., Kereszturi E., Toth B., Csala M. (2020). Effect of cis- and trans-Monounsaturated Fatty Acids on Palmitate Toxicity and on Palmitate-induced Accumulation of Ceramides and Diglycerides. Int. J. Mol. Sci..

[6] Oteng A.B., Kersten S. (2020). Mechanisms of Action of trans Fatty Acids. Adv. Nutr..

[7] Brouwer I.A., Wanders A.J., Katan M.B. (2010). Effect of animal and industrial trans fatty acids on HDL and LDL cholesterol levels in humans—a quantitative review. PLoS ONE.

[8] Jacome-Sosa M., Vacca C., Mangat R., Diane A., Nelson R.C., Reaney M.J., Shen J., Curtis J.M., Vine D.F., Field C.J. (2016). Vaccenic acid suppresses intestinal inflammation by increasing anandamide and related N-acylethanolamines in the JCR:LA-cp rat. J. Lipid Res..

[9] He M.L., McAllister T.A., Kastelic J.P., Mir P.S., Aalhus J.L., Dugan M.E., Aldai N., McKinnon J.J. (2012). Feeding flaxseed in grass hay and barley silage diets to beef cows increases alpha-linolenic acid and its biohydrogenation intermediates in subcutaneous fat. J. Anim. Sci..

[10] Fontaine M.A., Diane A., Singh V.P., Mangat R., Krysa J.A., Nelson R., Willing B.P., Proctor S.D. (2019). Low birth weight causes insulin resistance and aberrant intestinal lipid metabolism independent of microbiota abundance in Landrace-Large White pigs. FASEB J..

[11] Lansing M., Turner J.M., Wizzard P., Lavallee C.M., Lim D.W., Muto M., Nation P.N., Pencharz P.B., Ball R.O., Wales P.W. (2019). Plasma citrulline is not a biomarker for intestinal adaptation in short bowel syndrome, studied in piglets: A model for human neonates. Pediatr. Surg. Int..

[12] Vahmani P., Rolland D.C., McAllister T.A., Block H.C., Proctor S.D., Guan L.L., Prieto N., Lopez-Campos O., Aalhus J.L., Dugan M.E.R. (2017). Effects of feeding steers extruded flaxseed on its own before hay or mixed with hay on animal performance, carcass quality, and meat and hamburger fatty acid composition. Meat Sci..

[13] Diane A., Borthwick F., Mapiye C., Vahmani P., David R.C., Vine D.F., Dugan M.E., Proctor S.D. (2016). Beef Fat Enriched with Polyunsaturated Fatty Acid Biohydrogenation Products Improves Insulin Sensitivity Without Altering Dyslipidemia in Insulin Resistant JCR:LA-cp Rats. Lipids.

[14] Han C., Vinsky M., Aldai N., Dugan M.E., McAllister T.A., Li C. (2013). Association analyses of DNA polymorphisms in bovine SREBP-1, LXRalpha, FADS1 genes with fatty acid composition in Canadian commercial crossbred beef steers. Meat Sci..

[15] Council N.R. (2012). Nutrient Requirements of Swine: Eleventh Revised Edition.

[16] Swindle M.M., Smith A.C. (2015). Swine in the Laboratory: Surgery, Anesthesia, Imaging, and Experimental Techniques.

[17] Uwiera R.R., Mangat R., Kelly S., Uwiera T.C., Proctor S.D. (2016). Long-Term Catheterization of the Intestinal Lymph Trunk and Collection of Lymph in Neonatal Pigs. J. Vis. Exp..

[18] Vine D.F., Takechi R., Russell J.C., Proctor S.D. (2007). Impaired postprandial apolipoprotein-B48 metabolism in the obese, insulin-resistant JCR:LA-cp rat: Increased atherogenicity for the metabolic syndrome. Atherosclerosis.

[19] Gossert A.D., Hinniger A., Gutmann S., Jahnke W., Strauss A., Fernandez C. (2011). A simple protocol for amino acid type selective isotope labeling in insect cells with improved yields and high reproducibility. J. Biomol. NMR.

[20] Lian J., Wei E., Groenendyk J., Das S.K., Hermansson M., Li L., Watts R., Thiesen A., Oudit G.Y., Michalak M. (2016). Ces3/TGH Deficiency Attenuates Steatohepatitis. Sci. Rep..

[21] Klindworth A., Pruesse E., Schweer T., Peplies J., Quast C., Horn M., Glockner F.O. (2013). Evaluation of general 16S ribosomal RNA gene PCR primers for classical and next-generation sequencing-based diversity studies. Nucleic Acids Res..

[22] Bolyen E., Rideout J.R., Dillon M.R., Bokulich N.A., Abnet C.C., Al-Ghalith G.A., Alexander H., Alm E.J., Arumugam M., Asnicar F. (2019). Reproducible, interactive, scalable and extensible microbiome data science using QIIME 2. Nat. Biotechnol..

[23] Callahan B.J., McMurdie P.J., Rosen M.J., Han A.W., Johnson A.J., Holmes S.P. (2016). DADA2: High-resolution sample inference from Illumina amplicon data. Nat. Methods.

[24] Katoh K., Misawa K., Kuma K., Miyata T. (2002). MAFFT: A novel method for rapid multiple sequence alignment based on fast Fourier transform. Nucleic Acids Res..

[25] McDonald D., Price M.N., Goodrich J., Nawrocki E.P., DeSantis T.Z., Probst A., Andersen G.L., Knight R., Hugenholtz P. (2012). An improved Greengenes taxonomy with explicit ranks for ecological and evolutionary analyses of bacteria and archaea. ISME J..

[26] Bokulich N.A., Kaehler B.D., Rideout J.R., Dillon M., Bolyen E., Knight R., Huttley G.A., Gregory Caporaso J. (2018). Optimizing taxonomic classification of marker-gene amplicon sequences with QIIME 2’s q2-feature-classifier plugin. Microbiome.

[27] Myrie S.B., McKnight L.L., King J.C., McGuire J.J., Van Vliet B.N., Cheema S.K., Bertolo R.F. (2017). Intrauterine growth-restricted Yucatan miniature pigs experience early catch-up growth, leading to greater adiposity and impaired lipid metabolism as young adults. Appl. Physiol. Nutr. Metab..

[28] Wang Y., Lu J., Ruth M.R., Goruk S.D., Reaney M.J., Glimm D.R., Vine D.F., Field C.J., Proctor S.D. (2008). Trans-11 vaccenic acid dietary supplementation induces hypolipidemic effects in JCR:LA-cp rats. J. Nutr..

[29] Jacome-Sosa M.M., Lu J., Wang Y., Ruth M.R., Wright D.C., Reaney M.J., Shen J., Field C.J., Vine D.F., Proctor S.D. (2010). Increased hypolipidemic benefits of cis-9, trans-11 conjugated linoleic acid in combination with trans-11 vaccenic acid in a rodent model of the metabolic syndrome, the JCR:LA-cp rat. Nutr. Metab..

[30] Wang Y., Jacome-Sosa M.M., Ruth M.R., Goruk S.D., Reaney M.J., Glimm D.R., Wright D.C., Vine D.F., Field C.J., Proctor S.D. (2009). Trans-11 vaccenic acid reduces hepatic lipogenesis and chylomicron secretion in JCR:LA-cp rats. J. Nutr..

[31] Duval C., Muller M., Kersten S. (2007). PPARalpha and dyslipidemia. Biochim. Biophys. Acta.

[32] Wang Y., Jacome-Sosa M.M., Ruth M.R., Lu Y., Shen J., Reaney M.J., Scott S.L., Dugan M.E., Anderson H.D., Field C.J. (2012). The intestinal bioavailability of vaccenic acid and activation of peroxisome proliferator-activated receptor-alpha and -gamma in a rodent model of dyslipidemia and the metabolic syndrome. Mol. Nutr. Food Res..

[33] Barter P.J. (2013). High density lipoprotein: A therapeutic target in type 2 diabetes. Endocrinol. Metab..

[34] Bermudez B., Lopez S., Ortega A., Varela L.M., Pacheco Y.M., Abia R., Muriana F.J. (2011). Oleic acid in olive oil: From a metabolic framework toward a clinical perspective. Curr. Pharm. Des..

[35] Tholstrup T. (2006). Dairy products and cardiovascular disease. Curr. Opin. Lipidol..

[36] Tricon S., Burdge G.C., Jones E.L., Russell J.J., El-Khazen S., Moretti E., Hall W.L., Gerry A.B., Leake D.S., Grimble R.F. (2006). Effects of dairy products naturally enriched with cis-9,trans-11 conjugated linoleic acid on the blood lipid profile in healthy middle-aged men. Am. J. Clin. Nutr..

[37] Parodi P.W. (2016). Cooperative action of bioactive components in milk fat with PPARs may explain its anti-diabetogenic properties. Med. Hypotheses.

[38] Musso G., Gambino R., De Michieli F., Cassader M., Rizzetto M., Durazzo M., Faga E., Silli B., Pagano G. (2003). Dietary habits and their relations to insulin resistance and postprandial lipemia in nonalcoholic steatohepatitis. Hepatology.

[39] Costello E.K., Carlisle E.M., Bik E.M., Morowitz M.J., Relman D.A. (2013). Microbiome assembly across multiple body sites in low-birthweight infants. MBio.

[40] Pedersen R., Andersen A.D., Molbak L., Stagsted J., Boye M. (2013). Changes in the gut microbiota of cloned and non-cloned control pigs during development of obesity: Gut microbiota during development of obesity in cloned pigs. BMC Microbiol..

[41] Pedersen R., Andersen A.D., Hermann-Bank M.L., Stagsted J., Boye M. (2013). The effect of high-fat diet on the composition of the gut microbiota in cloned and non-cloned pigs of lean and obese phenotype. Gut Microbes.

[42] Panasevich M.R., Meers G.M., Linden M.A., Booth F.W., Perfield J.W., Fritsche K.L., Wankhade U.D., Chintapalli S.V., Shankar K., Ibdah J.A. (2018). High-fat, high-fructose, high-cholesterol feeding causes severe NASH and cecal microbiota dysbiosis in juvenile Ossabaw swine. Am. J. Physiol. Endocrinol. Metab..

[43] Fouhse J., Yang K., Li J., Mills E., Ju T., Alvarado C.S., Chan C.B., Willing B.P. (2018). Establishing a model for childhood obesity in adolescent pigs. Obes. Sci. Pract..

[44] Al K., Sarr O., Dunlop K., Gloor G.B., Reid G., Burton J., Regnault T.R. (2017). Impact of birth weight and postnatal diet on the gut microbiota of young adult guinea pigs. PeerJ.

[45] O’Donovan A.N., Herisson F.M., Fouhy F., Ryan P.M., Whelan D., Johnson C.N., Cluzel G., Ross R.P., Stanton C., Caplice N.M. (2020). Gut microbiome of a porcine model of metabolic syndrome and HF-pEF. Am. J. Physiol. Heart Circ. Physiol..

[46] Sze M.A., Schloss P.D. (2016). Looking for a Signal in the Noise: Revisiting Obesity and the Microbiome. mBio.

[47] Huang E.Y., Leone V.A., Devkota S., Wang Y., Brady M.J., Chang E.B. (2013). Composition of dietary fat source shapes gut microbiota architecture and alters host inflammatory mediators in mouse adipose tissue. JPEN J. Parenter. Enter. Nutr..

[48] Lam Y.Y., Ha C.W., Hoffmann J.M., Oscarsson J., Dinudom A., Mather T.J., Cook D.I., Hunt N.H., Caterson I.D., Holmes A.J. (2015). Effects of dietary fat profile on gut permeability and microbiota and their relationships with metabolic changes in mice. Obesity.

[49] Caesar R., Tremaroli V., Kovatcheva-Datchary P., Cani P.D., Backhed F. (2015). Crosstalk between Gut Microbiota and Dietary Lipids Aggravates WAT Inflammation through TLR Signaling. Cell Metab..

