# Supplementary material for: High Vaccenic Acid Content in Beef Fat Attenuates High Fat and High Carbohydrate Western Diet Induced Changes in Lipid Metabolism and Gut Microbiota in Pigs
Source: Microorganisms. 2021 Dec 6;9(12):2517. doi: 10.3390/microorganisms9122517 (PMC8707829; doi:10.3390/microorganisms9122517)
Supplement: Supplementary file 1 [file microorganisms-09-02517-s001.zip › microorganisms-1310334-supplementary.pdf]

Supplementary Materials

Table S1. Equations for the (Appearance 0-30 min) and clearance (30-120 min) phases of the postprandial glucose insulin and triglyceride curves. * denotes significance compared to LBW-CD, ** denotes significance compared to IR-HFHC-LVA

Groups	Glucose		Insulin		Triglyceride	
	**Appearance (0-30 min)**	**Clearance (30–120 min)**	**Appearance (0–30 min)**	**Clearance (30–120 min)**	**Appearance (0–30 min)**	**Clearance (30–120 min)**
**Control-CD**	0.174x + 5.60	−0.0484x + 10.80	0.551x + 2.08	−0.166x + 19.30	0.0439x + 3.39	0.0022x + 7.89
**LBW-CD**	0.136x + 5.35	−0.043x + 9.59	0.605x + 2.13	−0.182x + 20.60	0.0665x + 4.43	0.00442x + 12.50
**IR-HFHC-LVA**	0.257x + 4.96*	−0.0729x + 15.30	1.06x + 1.80*	−0.354x +44.70*	0.0756x + 12.40	−0.0555x + 26.50 *
**IR-HFHC-HVA**	0.198x + 4.78	−0.0668x + 13.30	0.58x + 18**	−0.199x + 25.40**	-0.0168x + 11.50**	0.00459x + 8.43**

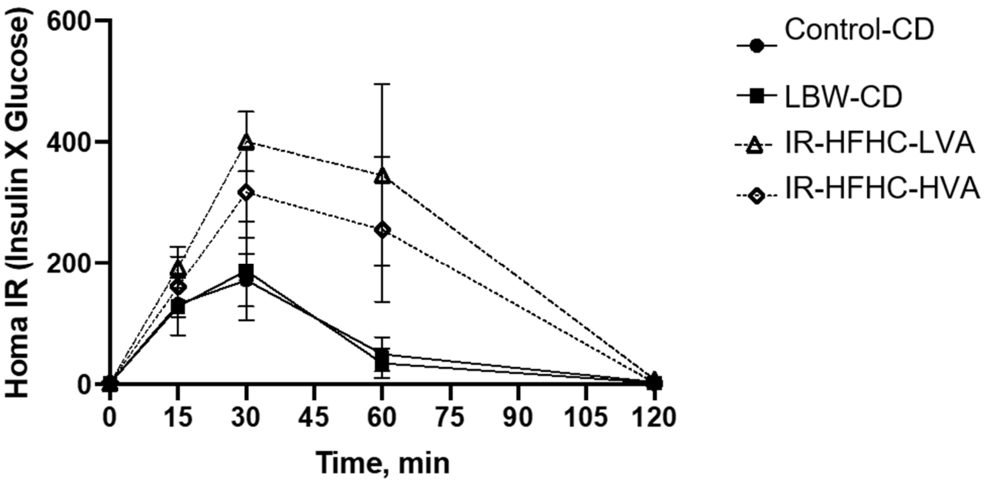

Figure S1. Homeostatic Model of Assessment of Insulin Resistance (HOMA IR). Assessed by product of postprandial blood glucose and plasma insulin concentration for determination of insulin resistance.

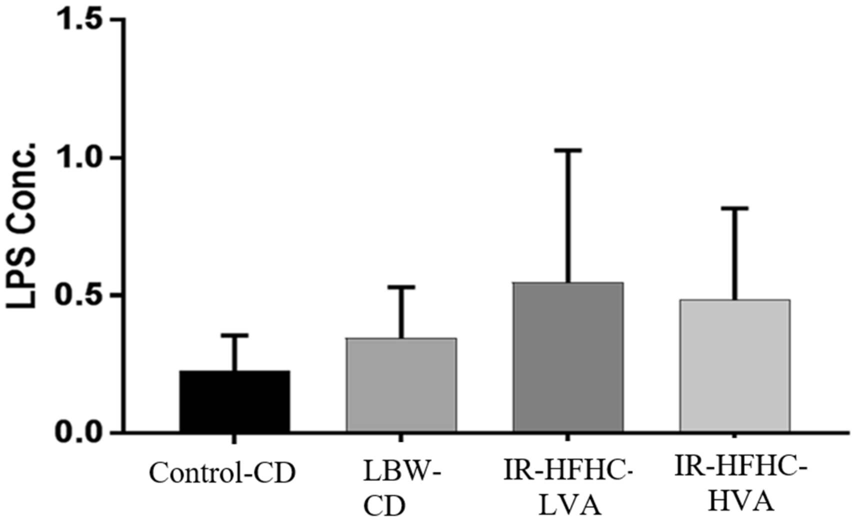

Figure S2. Concentration of serum lipopolysaccharide (LPS) in control and LBW pigs on control diet, HFHC-LVA or HFHC-HVA diet. HFHC-HVA did not affect LPS in IR swine. Values are means ± SD. CD, control diet; IR, Insulin resistant; HFHC, high-fat, high-carbohydrate diet; HFHC-HVA, high-fat, high-carbohydrate diet enriched with vaccenic acid, (p = 0.852).
